# Supplementary figures and images for: Effect of Proton Therapy on Tumor Cell Killing and Immune Microenvironment for Hepatocellular Carcinoma
Source: Cells. 2023 Jan 15;12(2):332. doi: 10.3390/cells12020332 (PMC9857172; doi:10.3390/cells12020332)

# Supplementary Figure S1

(a)

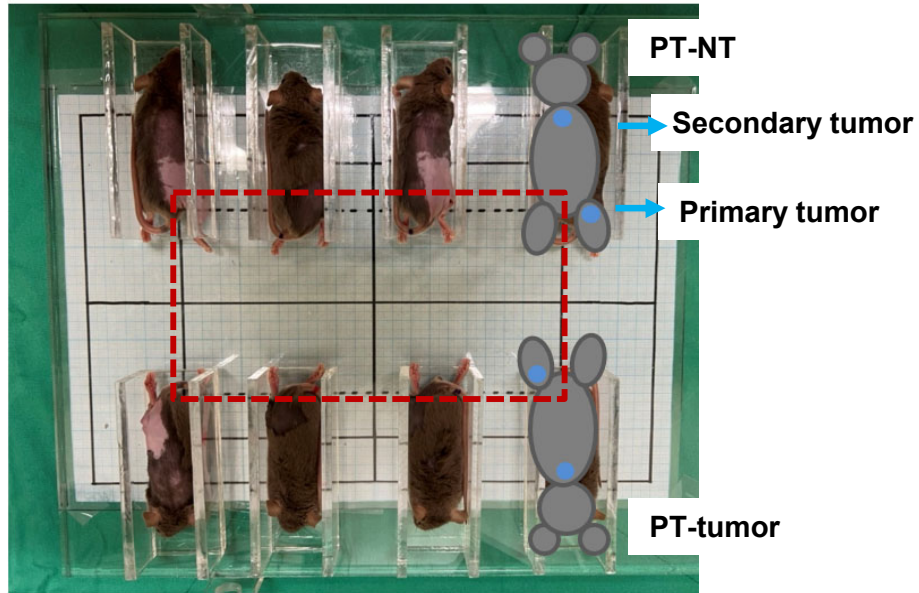

(c) Secondary tumor

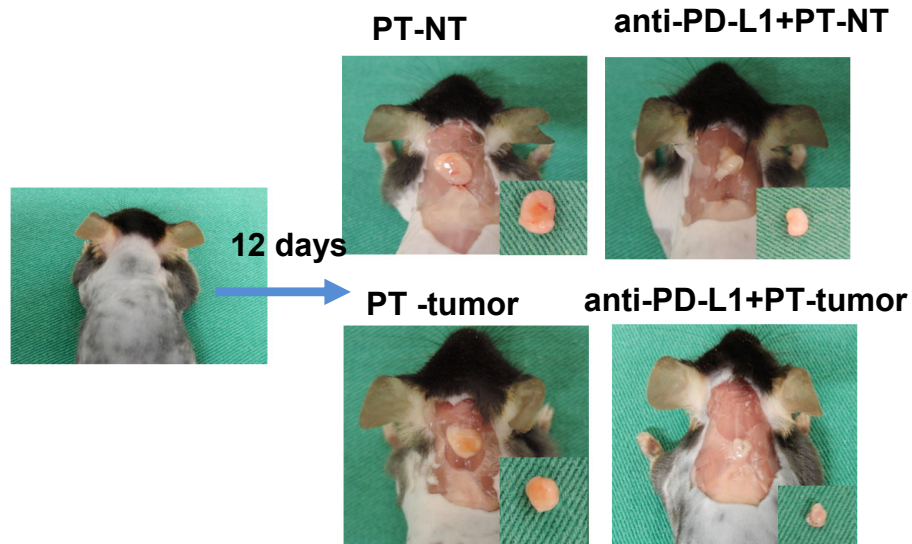

(b)

PT-NT

Anti-PD-L1  
+PT-NT

PT-tumor

Anti-PD-L1  
+PT-tumor

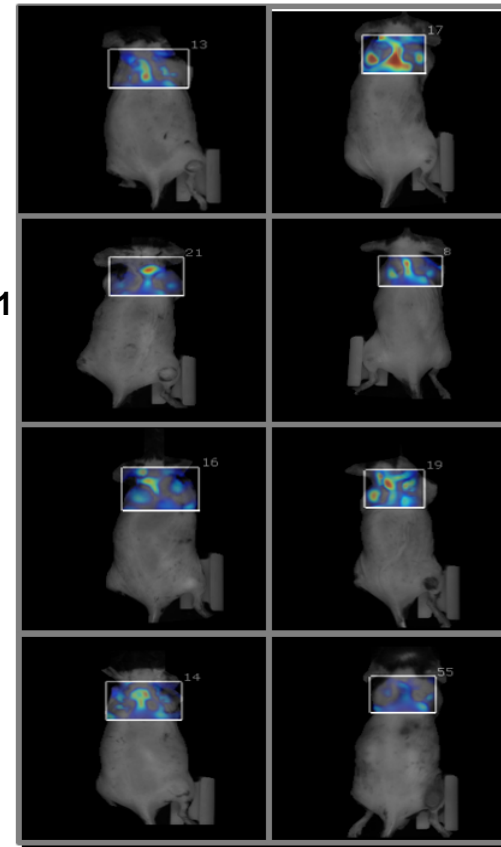

Supplement: Supplementary file 1 [file cells-12-00332-s001.zip › cells-2078711-supplementary.pdf]
